# Intelligent Transmissive Microwave Metasurface with Optical Sensing and Transparency

**DOI:** 10.34133/research.0514

**Published:** 2024-10-21

**Authors:** Ya Lun Sun, Xin Ge Zhang, Zhixiang Huang, Han Wei Tian, Tie Jun Cui, Wei Xiang Jiang

**Affiliations:** ^1^ State Key Laboratory of Millimeter Waves, School of Information Science and Engineering, Southeast University, Nanjing 210096, China.; ^2^ Information Materials and Intelligent Sensing Laboratory of Anhui Province, Anhui University, Hefei 230039, China.; ^3^ Institute of Electromagnetic Space, Southeast University, Nanjing 210096, China.; ^4^ Purple Mountain Laboratories, Nanjing 211111, China.

## Abstract

Transmissive metasurfaces are essentially conducive to stealth, absorbers, and communications. However, most of the current schemes only allow microwave to transmit and generally adopt multilayer structures or thick dielectric substrates to improve the electromagnetic performance, restricting optical transmission and conformal application. In addition, most metasurfaces still require metal wires and external power suppliers for programmability. Here, we propose and design an intelligent transmissive microwave metasurface with optical sensing and transparency, which provides both microwave and optical channels without redundant optical devices and power suppliers, and the 2 transmission channels are associated with each other. The metasurface is realized by validly integrating photosensitive materials into microwave meta-structures. As a demonstration, we fabricate an ultrathin optically transparent transmissive metasurface based on polyethylene terephthalate substrate and photoresistors, whose thickness is only 0.125 mm. We further construct cross-wavelength transmission links based on the metasurface sample and experimentally validate that the microwave transmissions vary with light intensities under full-polarization and large-angle incidences, and this metasurface possesses high optical transparency. The intelligent transmissive microwave metasurface with optical sensing and transparency has potential applications in optical–microwave hybrid transmission devices and stealth technology.

## Introduction

Metasurfaces are ultrathin artificially manufactured 2-dimensional (2D) metamaterials and are generally made of metal conductors and dielectric substrates in the early days [1]. Due to their unique capabilities in manipulating electromagnetic (EM) waves to realize various particular functions, they have acquired extensive research interests. By regularly arranging elaborately designed metasurface units in array, metasurface is able to tune the amplitude, phase, and frequency of EM waves conveniently and rapidly [2–7]. Furthermore, digital and programmable metasurfaces are capable of reconfiguring EM waves in real time [8–14], which have demonstrated great freedom in EM manipulation. In advanced information transmission, EM stealth, and electronic countermeasures, metasurface gradually reveals its distinctive capabilities, especially the stealth with transmission window [15–17]. Nevertheless, vast majority of these metasurfaces are opaque substantially and the transmitted wave is microwave, which seriously restricts the efficient utilization of optical wavelength.

In reality, capabilities of visibility and transparency are important for EM devices, because of the supplementary window for optical transmission. Thus, numerous attempts have been made in optically transparent metasurfaces, which have great potentials in optical and EM stealth, integrated communications, absorbers, and lens [18–28]. Recently, several optically transparent materials have been explored for EM metasurface, such as indium tin oxide, silver nanowires, polycarbonate, and polyethylene terephthalate (PET) [26–33]. However, the EM performance and optical transparency are mutually restricted [34,35], leading to the low light transmittance of transmissive metasurfaces with complicated EM features. Nowadays, in addition to transparency, the adjustability and reconfigurability of metasurfaces appear more and more important gradually, like the graphene-based [36] and varactor-integrated [37,38] optically transparent metasurfaces. These metasurfaces employ transparent materials as dielectric substrate and require metal wires and external direct current (DC) voltage sources to provide bias signals for integrated graphene and varactor, which will interfere with the EM performance of the metasurface and increase power dissipation. To remove the redundant DC voltage sources and realize low-power metasurfaces, researchers innovatively integrated photoelectric devices into metasurfaces to achieve optical–microwave coupling [39–42], but these metasurfaces are unable to realize optical transmission. Thus, how to realize low-power EM metasurfaces with flexible microwave manipulation and optical transmission still remains challenging.

In this article, we propose and realize an intelligent transmissive microwave metasurface with optical sensing and transparency, which can supply an adaptive light-dependent microwave channel and a flexible optical channel simultaneously. The microwave transmission amplitude of the metasurface can be remotely tuned by light in real time, and high optical transmission and physical flexibility can be achieved since the metasurface is designed by etching out thin copper wires on the surface of PET dielectric substrate. To realize the light-controllable microwave transmissions, each metasurface element is integrated with photosensitive materials with tunable impedance, thus affecting the microwave transmission level. The photosensitive transmissive metasurface element is able to perceive light and tune microwave simultaneously. Therefore, the microwave transmission amplitude of the metasurface can be tuned by light, without redundant optical devices and power supply. Besides, the metasurface adopts single-layer ultrathin PET and the thickness is only 0.125 mm, with good conformal properties, easy to be attached to windows, walls, and other objects.

## Results and Discussion

**Fig. 1.**
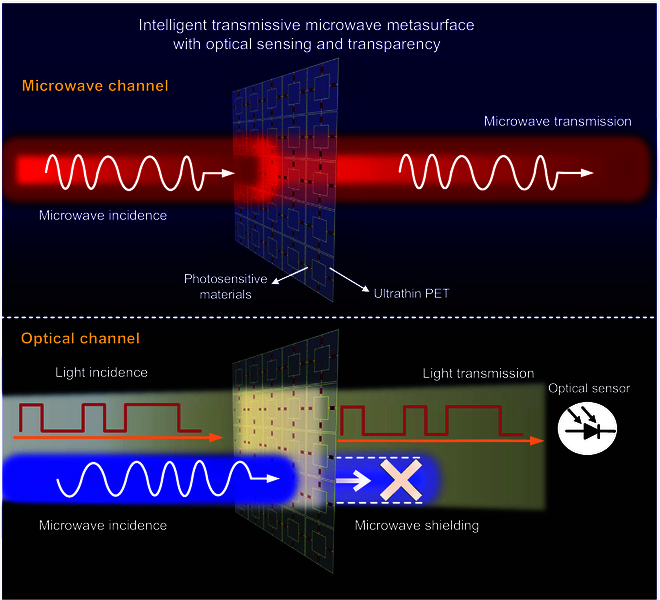
Illustration of the proposed intelligent transmissive microwave metasurface with optical sensing and transparency. The intelligent transmissive microwave metasurface has adaptively light-dependent microwave and flexible optical channels. It is realized by integrating photosensitive materials into microwave meta-structure, which can respond to the incident optical and microwave fields simultaneously. Therefore, the transmission microwave amplitude of this metasurface is able to be tuned by light dynamically, without redundant optical devices and power suppliers, realizing light-dependent microwave transmission and optical transmission.

The intelligent transmissive microwave metasurface with optical sensing and transparency is shown in Fig. 1, and such a metasurface has adaptively light-dependent microwave and flexible optical channels. It can transmit not only microwaves, but also optical waves, realizing cross-wavelength information transmission. Moreover, the microwave transmission depends on optical transmission, because the metasurface is sensitive to light intensity and can regulate the microwave transmission amplitude by dynamic light adaptively. Thus, when the illuminating light is closed, the microwave channel operates well. While illuminated by strong light, the optical channel works, and the microwave channel will be stopped adaptively. On the microwave channel, this metasurface allows microwave transmission with optical transparency. However, when it is necessary to avoid radar detection or protect internal electronic components from EM damage, the metasurface allows light transmission for optical communication, optical energy transmission, and illumination; meanwhile, it can realize microwave shielding automatically. The metasurface unit is composed of a microwave resonator structured to respond to microwaves and photosensitive materials such as photoresistors in response to the light intensity, in which the impedance changes by light; subsequently, the resonance state of the metasurface unit is different, thus tuning microwave amplitudes. This material integration is simultaneously responds to the incident optical and microwave fields, realizing dynamically manipulating transmission amplitude by light on a single-layer metasurface without additional optical devices or power components. The distinctively designed metasurface unit is able to tune amplitudes under full polarization and large angle incidence. Moreover, this metasurface is manufactured by transparent thin materials as substrates, to obtain properties of optical transparency and flexibility. Therefore, the optically transparent and flexible transmissive metasurface film with light-dependent microwave and optical channels is able to be covered on windows and other non-planar objects without affecting visibility.

Importantly, compared with traditional transmissive metasurface, our intelligent transmissive microwave metasurfaces with optical sensing and transparency provide more diverse transmission windows including microwave and optical channels, which promises to bring more advantages in EM countermeasures. In addition, the switch of microwave channel is dependent on optical channel, and transmission amplitudes can be tuned in real time completely and directly by light wirelessly, without redundant optical devices, power supplies, or complex control circuits. Besides, due to the optical control method and unique metasurface structure, the metasurface can adopt a single-layer ultrathin dielectric substrate instead of a multilayer structure with via holes, with good conformal properties and high optical transparency. Therefore, our metasurface has great potentials in cross-wavelength transmission and remote EM regulation, and provides a new approach for constructing optical microwave hybrid devices.

**Fig. 2.**
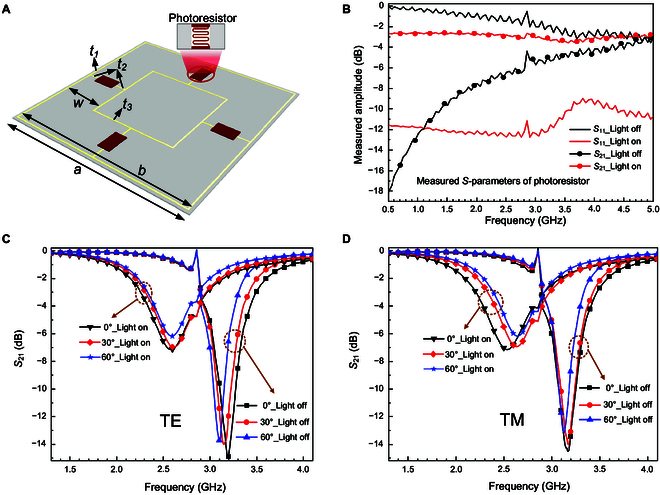
The structure and simulation results of the metasurface unit and measured *S*-parameters of the photoresistor. (A) Overall view of the metasurface unit; 4 identical photoresistors are arranged central symmetrically between 2 metal concentric square rings. (B) The measured *S*_11_ and *S*_21_ curves of the used “SG3624” photoresistor. Simulated transmission amplitudes of the designed metasurface unit for 2 different states (“light on” and “light off”) under (C) TE and (D) TM polarized microwave incidence of several different angles like 0°, 30°, and 60°, respectively.

To achieve good optical transmission and flexibility based on PET as the substrate, the photosensitive transmissive metasurface unit is mainly designed by using ultrathin and fine copper wires, as shown in Fig. 2A. To realize good polarization insensitivity, we adopt an symmetric square structure to implement the metasurface unit, in which there are 2 concentric square rings made of copper wires. Between the 2 metal square rings, 4 identical photoresistors are arranged central symmetrically, connected to the sides of meta square rings by metal short wires, and the distance from the center of the photoresistor to the 2 contiguous metal rings is the same. This symmetric geometry contributes to high polarization insensitivity. To ensure fine flexibility and transparency, the thickness of PET (dielectric constant 3.00, loss tangent 0.06) and copper wire is 0.125 and 300 nm, respectively. The width of all copper wires is smaller than 0.3 mm, to reduce the impact on light penetrability as much as possible, without destroying microwave functions of metasurface. Additionally, to achieve the wide-angle characteristic, miniaturizing metasurface unit is a feasible solution, which has been verified in previous works [43,44]. According to this miniaturized-unit scheme to realize angle insensitivity, we design a small-dimension metasurface unit (only 0.26 λ at 2.6 GHz). The subsequent simulation results demonstrate that our proposed metasurface unit has angle insensitivity with a maximum incidence angle of 60°. The specific parameters of the metasurface unit are determined through simulation and optimization, to achieve the optimal manipulation of microwave transmission. The specific parameters are optimally determined as *a* = 30.0 mm, *b* = 29.0 mm, *w* = 7.0 mm, *t*_1_ = 0.15 mm, *t*_2_ = 0.2 mm, *t*_3_ = 0.25 mm (*t*_1_, *t*_2_, and *t*_3_ indicate the width of copper wires). These integrated photoresistors are adopted as microwave tunable components and light perception simultaneously. Through such integration of microwave unit and photosensitive materials, we can reach the light-adaptive microwave regulation directly without extra voltage sources, optical components, or photoelectric conversion.

The metasurface unit is simulated in the software CST Microwave Studio, in which the scattering parameters of photoresistor we used are experimentally measured. Under the illuminations of light, the resistance of photoresistor reduces to about 50 Ω from 200 kΩ, which tunes the transmission coefficients (*S*_21_) of the metasurface unit. When the light is turned off, the resistance of the photoresistor is 200 kΩ, and then the microwave will be transmitted at the designed frequency band. When the light is turned on, the resistance of the photoresistor is 50 Ω, and then the microwave is hard to transmit due to impedance mismatch. However, at this state, the light can pass through well due to the high transparency property of the metasurface. In this case, the transmission states of the microwave channel and optical channel are opposite. According to these measured scattering parameters of the used photoresistor for state “light on” and “light off” (see Fig. 2B), in the unit cell boundary condition, the simulation is conducted and results are presented in Fig. 2C and D. Due to the centrosymmetric structure of the photosensitive transmissive metasurface unit, it is able to regularly operate under the irradiation of full-polarization microwave. Figure 2C shows the *S*_21_ curves under the TE polarized incidence of several different angles like 0°, 30°, and 60°, respectively. While the angle is 0°, microwaves are vertically irradiated on the metasurface, and the amplitude differences of *S*_21_ between 2 states are 7 dB at 2.6 GHz and 13 dB at 3.2 GHz. The *S*_21_ curves under the TM polarized incidence shown in Fig. 2D are basically consistent with that under the TE polarized incidence. Theoretically, at high frequencies, the photoresistor also has parasitic capacitance variable by light with resistance. Hence, we substitute the scattering parameters measured at high frequencies into the simulation of the metasurface unit, instead of elementary resistor–inductor–capacitor equivalent circuits. Therefore, the resonant peak frequency point of “light on” and “light off” is different, shifting from 2.6 to 3.2 GHz, meaning that the resonance state of the metasurface unit can be changed by light. During 1.5 and 2.91 GHz, the *S*_21_ of state “light off” is greater than “light on”, and it is the opposite when the frequency is above 2.91 GHz. In the range of 2.28 to 2.87 GHz and 3 to 3.4 GHz, the amplitude differences of *S*_21_ are larger than 3 dB. Furthermore, the elaborately designed metal square rings of the metasurface unit can certainly contain the angular dispersion; thus, this metasurface has a wide incidence angle and the maximum angle reaches 60°. It is obviously indicated that resonant frequency points shift slightly with incidence angle increasing. Overall, under a 0° to 60° incidence angle, the *S*_21_ differences are substantially similar at frequencies near 2.6 and 3.2 GHz. Therefore, this proposed photosensitive transmissive metasurface unit indeed has capabilities of responding to full-polarization microwaves with wide incidence angles and tuning transmission amplitudes by light simulatively.

**Fig. 3.**
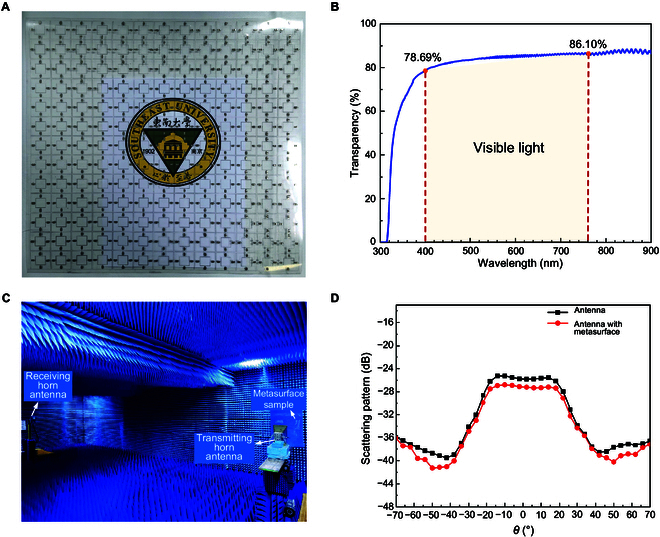
Realization of the proposed metasurface and measured far-field patterns. (A) The fabricated optically transparent and flexible transmissive metasurface sample based on PET substrate and composed of photoresistors. (B) The measured light transmittance rate of metasurface sample. (C) Experimental setup in the anechoic chamber. (D) The measured far-field patterns of antenna without and with metasurface sample (“light off” state) at 2.6 GHz.

Based on the realized photosensitive transmissive metasurface unit, we construct the metasurface to further explore its optical and microwave features. Comprehensively considering actual manufacturing and microwave performance, we arranged 13 × 13 metasurface units into a square array as the metasurface. By etching metal patterns on pet substrates and soldering photoresistors onto metal wires, this optically transparent and flexible metasurface sample was fabricated. The model of photoresistor is SG3624, which is surface-mount component packaging, with a size of 2.4 mm × 3.6 mm. The size of this metasurface sample is 390 mm × 390 mm. As shown in Fig. 3A, the whole metasurface is practically optically transparent except for photoresistors, and it is an ultrathin film, so we can see the picture under the metasurface clearly. To accurately demonstrate the transparency of the metasurface, we measured the light transmittance rate under different wavelengths from 300 to 900 nm by using the Shimadzu UV-VIS spectrophotometer UV2600 (see Note S1 for the experiment on optical transmittance). This spectrophotometer is a commonly used instrument for accurately measuring the transmission or absorption of a sample in different spectra. In measurement, the sample needs to be placed in the sample chamber and covered with a lid to prevent interference of external light, and the experiment should be conducted in a dry environment for accurate measurement. Due to limited space of the sample chamber that requires the area of the measured film to be within 4 cm × 4 cm generally, a metasurface unit sample was cut out to be measured instead of the whole sample. The unit sample was placed vertically on the optical path to reduce optical reflection and then the transmittance within the configurated spectral range was scanned and plotted as a curve. The measured results are shown in Fig. 3B. This metasurface has a high optical transparency of about 80% over the whole visible wavelength spectrum from 400 to 760 nm, basically not obstructing the normal transmission of visible light, because of the distinctively designed single-layer dielectric substrate and slender metal wires. According to such results, the metasurface is verified to have high light transparency, and as microwave devices, its impacts on antennas are worth considering as well. Thus, we set up experiments in the anechoic chamber to measure the influence of this metasurface on antennas. The metasurface sample is placed 0.9 m in front of the transmitting horn antenna, while the receiving horn antenna is located far away, as shown in Fig. 3C. Figure 3D shows the measured far-field patterns without and with the metasurface sample (“light off” state), respectively, at 2.6 GHz, and it is obvious that the patterns of 2 cases are hardly different except for a loss of 1.4 dB caused by the metasurface sample. Therefore, this metasurface has satisfactory optical and microwave transmission performance. On the other hand, this metasurface can be attached to other objects, intentionally transforming them to be light-dependent microwave tunable objects conveniently, approximately without changing the original visibility and microwave performance.

**Fig. 4.**
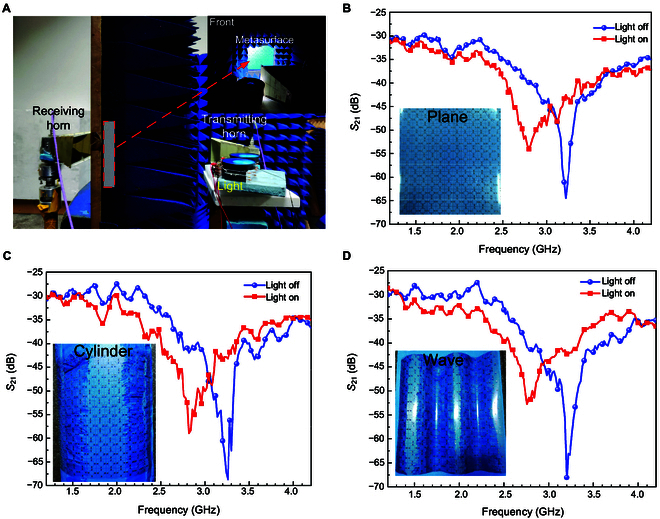
Experimental setup and measurement results of the intelligent transmissive microwave metasurface sample. (A) Photograph of the experimental setup. The measured transmission amplitudes curves of the metasurface sample stuck on the surface of (B) the plane model, (C) the cylinder model, and (D) the wave model, respectively, for 2 states.

As experimental verifications of the adaptively light-dependent microwave transmission channel, we further established a measurement scenario, as shown in Fig. 4A. There is a couple of horn antennas placed on different sides of the metasurface sample, connected to the vector network analyzer as receiving and transmitting, respectively, with the same height and linear polarization. From the front view inset, we can observe that the metasurface sample is fixed on the absorbing surface with a rectangular window to transmit EM waves, to avoid measured error caused by interferences of irrelevant microwaves. The distance between metasurface and 2 antennas is 1 m separately. The state is “light on” when the metasurface sample is illuminated by the light source and, conversely, “light off” without light. Additionally, to further verify its EM properties under wide angle incidence, we manufactured a cylinder model (shown in Fig. 4C) with a radius of 21 cm and a wave model (shown in Fig. 4D) made of foam with a dielectric constant of around 1. Then, the metasurface sample was stuck on the surface of the cylinder model and wave model, respectively; in this situation, the maximum incidence angle can reach 60°. Thus, the wide-angle incidence performance can also be measured when the transmitting antenna irradiates on the metasurface vertically, and it is able to demonstrate the practical capability when conforming on complex surfaces.

The measured results are shown in Fig. 4B to D. According to the measured *S*_21_ curves of the metasurface stuck on the plane surface in Fig. 4B, we can investigate that the frequency point of the resonant peak about “light on” and “light off” is 2.78 and 3.22 GHz, respectively, substantially consistent with simulation results. The amplitude differences of *S*_21_ at the 2 frequency points are approximately 10 and 15 dB, respectively. From 2.48 to 3.02 GHz, the *S*_21_ of state “light off” is larger than that of state “light on”, while during 3.16 and 3.38 GHz, the *S*_21_ of state “light on” is greater in contrast, and within the 2 frequency bands, the transmission amplitude differences between 2 states are beyond 3 dB. As to the metasurface sample conformed on the cylinder model and wave model, the measured *S*_21_ curves are similar to that of the plane model, indicating the effective angular stability. Besides, the actual manufactured square rings in the metasurface composed of ultrathin slender copper wires introducing additional resistances and the position of metasurface and antennas both affect the measured results. On the other hand, each photoresistor integrated in the metasurface may not be completely identical on resistance and required light intensity, causing certain influences. Consequently, the proposed photosensitive transmissive metasurface is able to respond to full-polarization microwaves with wide incidence angles and tune microwave transmission amplitudes by light adaptively.

**Fig. 5.**
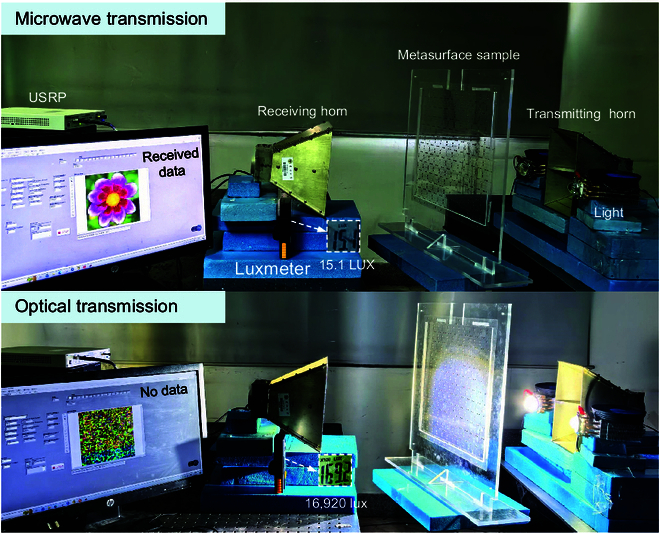
Photograph of the cross-wavelength transmission links with adaptively light-dependent microwave and flexible optical channels based on the metasurface sample. When light source is off, microwave with data can transmit through metasurface; when light source is on, microwave is shielded, and light transmits efficiently.

To further demonstrate the potential function of this intelligent transmissive microwave metasurface with adaptively light-dependent microwave and flexible optical channels, we structured a cross-wavelength transmission link based on the metasurface sample, as shown in Fig. 5. The transmitter is composed of a microwave transmitting horn antenna and light source, while the receiver consists of a microwave receiving horn antenna and a universal software radio peripheral (USRP) with a computer, as well as a luxmeter to measure the received light intensity. The metasurface sample is emplaced between the transmitter and the receiver. The operating frequency of the microwave channel in this transmission link is configured as 2.78 GHz. When the light is off (the received light intensity is 15.1 lux), the data (a flower picture) transmitted from the microwave transmitting horn can be received by the receiving horn and displayed on the screen, in which the microwave channel can operate successfully. When the light is on, the data cannot be received due to the microwave shielding by the metasurface, and the received light intensity is 16,920 lux, realizing the high-efficient optical transmission. In this cross-wavelength transmission link, the microwave channel is adaptively dependent on the optical channel, while the optical channel is flexible, in which light is used to tune microwave and transmitted simultaneously. In this experiment, we use the on and off switching of light to tune the microwave channel and demonstrate the use of the optical channel for illumination with high-efficient optical transmission. When using rapidly varying light intensity signals, the optical channel remains open, and the microwave channel of the metasurface can still remain the closed state (Fig. S1), without affecting the microwave shielding function (see Note S2 for measurement of the response time of the metasurface). Due to the channel stability of our metasurface under high-speed varying light, we can use the optical channel to transmit information based on varying light intensity signals, with microwave shielding. As shown in Fig. S2, we conducted experiments of using the optical channel to transmit information. The optical information transmission module composed of the field programmable gate array, the digital-to-analog converter, and a light source is located on the right side of the metasurface sample. The receiving module is located on the left of the metasurface sample, composed of a photoelectric detection circuit and an oscilloscope. Through the optical channel of the metasurface, the data signals composed of multiple waveforms can be received and then recovered to digital information (see Note S3 for experiments using an optical channel to transmit information). In normal states, the microwave channel operates, allowing normal radio frequency communication. When it is necessary to avoid EM detection and destruction, the optical channel operates and the microwave channel is shielded adaptively; thus, we can alternatively use strong light to transmit information, supply energy for devices, and illuminate, without being severely affected by electronic countermeasures.

These application scenarios demonstrated above use the on and off switching of light to switch the state of the microwave channel, to realize intelligent microwave amplitude control. In addition, in certain applications, metasurfaces require the capability of quickly responding to varying light intensity for fast signal conversion and information transmission [6,7,39]. In this case, instead of photoresistors, photosensitive materials with high-speed response (such as Si, InGaAs, and CdTe) can be used in our metasurface, which is able to respond to rapidly varying light intensity signals; thus, it can be used for quick conversion of light to microwave and hybrid wireless communication. The photosensitive materials loaded on the metasurface can be selected according to usage scenarios. Therefore, our intelligent transmissive microwave metasurfaces with optical sensing and transparency can indeed be employed in cross-wavelength transmission and active EM shielding.

## Conclusion

We proposed and experimentally verified an intelligent transmissive microwave metasurface with optical sensing and transparency, which can tune the transmission amplitude by light dynamically, under full-polarization microwave incidence with wide angles, able to provide adaptively light-dependent microwave and flexible optical channels. The metasurface is conducted based on PET, and 4 photoresistors are integrated into each metasurface unit, which is able to change impedances according to light intensity, thus regulating microwave amplitudes and realizing the combination of microwave and photoelectricity. Composed of 13 × 13 metasurface units, we have presented and fabricated a practical transparent and flexible transmissive metasurface, and further constructed a cross-wavelength transmission link with light-dependent microwave and flexible optical channels based on the metasurface sample. Both measured and simulated results validate fine performances of this metasurface, and the transmission amplitude differences dependent on light intensity around 2.78 and 3.22 GHz exceed 9 dB, allowing a maximum conformal angle of 60°.

Compared to conventional transmissive metasurfaces, our proposed intelligent transmissive microwave metasurface with optical sensing and transparency can not only provide controllable microwave transmissions, but also provide extra optical transmission channel; more importantly, the microwave channel is dynamically associated with the optical channel. The metasurface is able to simultaneously respond to light and microwave without extra optical devices and power components, and the transmission amplitudes vary with light intensities flexibly. This metasurface shows special potentials in low-power optical and microwave hybrid devices and flexible optical sensor components. Moreover, this metasurface provides a new approach for intelligent EM stealth and multi-domain transmission.

## Data Availability

The data used to support the findings of this study are available from the corresponding authors upon request.

## Supplementary Materials

Supplementary 1 Notes S1 to S3 Figs. S1 and S2

## References

[1] Sievenpiper D, Zhang L, Broas RFJ, Alexopolous NG, Yablonovitch E. High-impedance electromagnetic surfaces with a forbidden frequency band. IEEE Trans Microw Theory Tech. 1999;47(11):2059–2074.

[2] Zhang XG, Sun YL, Yu Q, Cheng Q, Jiang WX, Qiu C-W, Cui TJ. Smart Doppler cloak operating in broad band and full polarizations. Adv Mater. 2021;33(17):e2007966. 33851447 10.1002/adma.202007966

[3] Zheng B, Ren H, An S, Tang H, Li H, Haerinia M, Dong Y, Fowler C, Zhang H. Tunable metasurface with dynamic amplitude and phase control. IEEE Access. 2021;9:104522–104529.

[4] Liao J, Guo S, Yuan L, Ji C, Huang C, Luo X. Independent manipulation of reflection amplitude and phase by a single-layer reconfigurable metasurface. Adv Opt Mater. 2022;10(4):2101551.

[5] Zhang L, Chen XQ, Liu S, Zhang Q, Zhao J, Dai JY, Bai GD, Wan X, Cheng Q, Castaldi G, et al. Space-time-coding digital metasurfaces. Nat Commun. 2018;9(1):4334. 30337522 10.1038/s41467-018-06802-0 PMC6194064

[6] Zhang XG, Sun YL, Zhu B, Jiang WX, Zhang Z, Cui TJ. Light-controllable time-domain digital coding metasurfaces. Adv Photonics. 2022;4(2): Article 025001.

[7] Zhang XG, Sun YL, Zhu B, Jiang WX, Yu Q, Tian HW, Qiu C-W, Zhang Z, Cui TJ. A metasurface-based light-to-microwave transmitter for hybrid wireless communications. Light Sci Appl. 2022;11(1):126. 35513383 10.1038/s41377-022-00817-5 PMC9072331

[8] Huang C, Sun B, Pan W, Cui J, Wu X, Luo X. Dynamical beam manipulation based on 2-bit digitally-controlled coding metasurface. Sci Rep. 2017;7(1):42302. 28176870 10.1038/srep42302 PMC5296720

[9] Zhang XG, Yu Q, Jiang WX, Sun YL, Bai L, Wang Q, Qiu C-W, Cui TJ. Polarization-controlled dual-programmable metasurfaces. Adv Sci. 2020;7(11):1903382. 10.1002/advs.201903382 PMC7284210 32537403

[10] Li L, Cui TJ, Ji W, Liu S, Ding J, Wan X, Bo Li Y, Jiang M, Qiu C-W, Zhang S. Electromagnetic reprogrammable coding-metasurface holograms. Nat Commun. 2017;8(1):197. 28775295 10.1038/s41467-017-00164-9 PMC5543116

[11] Zhang XG, Jiang WX, Cui TJ. Frequency-dependent transmission-type digital coding metasurface controlled by light intensity. Appl Phys Lett. 2018;113(9): Article 091601.

[12] Bai X, Zhang F, Sun L, Cao A, Zhang J, He C, Liu L, Yao J, Zhu W. Time-modulated transmissive programmable metasurface for low sidelobe beam scanning. Research. 2022;2022:9825903. 35928303 10.34133/2022/9825903 PMC9297726

[13] Cui TJ, Liu S, Bai GD, Ma Q. Direct transmission of digital message via programmable coding metasurface. Research. 2019;2019:2584509. 31549052 10.34133/2019/2584509 PMC6750087

[14] Sun YL, Zhang XG, Yu Q, Jiang WX, Cui TJ. Infrared-controlled programmable metasurface. Sci Bull. 2020;65(11):883–888. 10.1016/j.scib.2020.03.016 36747420

[15] Xu S, Dong FY, Guo WR, Han DD, Qian C, Gao F, Su WM, Chen H, Sun HB. Cross-wavelength invisibility integrated with various invisibility tactics. Sci Adv. 2020;6(39):eabb3755. 32967829 10.1126/sciadv.abb3755 PMC7531887

[16] Huang C, Yang J, Ji C, Yuan L, Luo X. Graphene-driven metadevice for active microwave camouflage with high-efficiency transmission window. Small Methods. 2021;5(2):e2000918. 34927886 10.1002/smtd.202000918

[17] Pang Y, Li Y, Qu B, Yan M, Wang J, Qu S, Xu Z. Wideband RCS reduction metasurface with a transmission window. IEEE Trans Antennas Propag. 2020;68(10):7079–7087.

[18] Zu HR, Wu B, Chen B, Li WH, Su T, Liu Y, Tang WX, He DP, Cui TJ. Optically and radiofrequency-transparent metadevices based on quasi-one-dimensional surface plasmon polariton structures. Nat Electron. 2023;6(7):525–533.

[19] Xia C, Lu Z, Zhang Y, Tan J. Broadband high optical transparent intelligent metasurface for adaptive electromagnetic wave manipulation. Research. 2024;7:0334. 38476476 10.34133/research.0334 PMC10927547

[20] Fan RH, Peng RW, Huang XR, Li J, Liu Y, Hu Q, Wang M, Zhang X. Transparent metals for ultrabroadband electromagnetic waves. Adv Mater. 2012;24(15):1980–1986. 22431279 10.1002/adma.201104483

[21] Zhang J, Shao L, Li Z, Zhang C, Zhu W. Graphene-based optically transparent metasurface capable of dual-polarized modulation for electromagnetic stealth. ACS Appl Mater Interfaces. 2022;14(27):31075–31084. 35770880 10.1021/acsami.2c04414

[22] Palmer SJ, Xiao X, Pazos-Perez N, Guerrini L, Correa-Duarte MA, Maier SA, Craster RV, Alvarez-Puebla RA, Giannini V. Extraordinarily transparent compact metallic metamaterials. Nat Commun. 2019;10(1):2118. 31073197 10.1038/s41467-019-09939-8 PMC6509127

[23] Li S, Tian M, Gao Q, Wang M, Li T, Hu Q, Li X, Wu Y. Nanometre-thin indium tin oxide for advanced high-performance electronics. Nat Mater. 2019;18(10):1091–1097. 31406368 10.1038/s41563-019-0455-8

[24] Li Y, Lin J, Guo H, Sun W, Xiao S, Zhou L. A tunable metasurface with switchable functionalities: From perfect transparency to perfect absorption. Adv Opt Mater. 2020;8(6):1901548.

[25] Ma Y, Wang J, Shi L, Xue S, Ran Y, Li J, Liu Y. Ultra-wideband, optically transparent, and flexible microwave metasurface absorber. Opt Mater Express. 2021;11(7):2206–2218.

[26] Li T, Chen K, Ding G, Zhao J, Jiang T, Feng Y. Optically transparent metasurface Salisbury screen with wideband microwave absorption. Opt Express. 2018;26(26):34384–34395. 30650861 10.1364/OE.26.034384

[27] Jiang RZ, Wu JW, Ma Q, Liang JC, Wu LJ, Zhou QY, Dai JY, Cheng Q, Cui TJ. Optically transparent metasurface with high RF transmittance and wide-angle stability for dual bands and dual polarizations. Adv Opt Mater. 2023;11(19):2300553.

[28] Kitayama D, Hama Y, Goto K, Miyachi K, Motegi T, Kagaya O. Transparent dynamic metasurface for a visually unaffected reconfigurable intelligent surface: Controlling transmission/reflection and making a window into an RF lens. Opt Express. 2021;29(18):29292–29307. 34615041 10.1364/OE.435648

[29] Ge J, Zhang Y, Li H, Dong H, Zhang L. Ultra-broadband, tunable, and transparent microwave meta-absorber using ITO and water substrate. Adv Opt Mater. 2023;11(10):2202873.

[30] Chen Z, Li W, Li R, Zhang Y, Xu G, Cheng H. Fabrication of highly transparent and conductive indium–tin oxide thin films with a high figure of merit via solution processing. Langmuir. 2013;29(45):13836–13842. 24117323 10.1021/la4033282

[31] Hu D, Cao J, Li W, Zhang C, Wu T, Li Q, Chen Z, Wang Y, Guan J. Optically transparent broadband microwave absorption metamaterial by standing-up closed-ring resonators. Adv Opt Mater. 2017;5(13):1700109.

[32] Deng G, Lv K, Sun H, Yang J, Yin Z, Chi B, Li X. An ultra-broadband and optically transparent metamaterial absorber based on multilayer indium-tin-oxide structure. J Phys D Appl Phys. 2021;54(16): Article 165301.

[33] Xu C, Wang B, Yan M, Pang Y, Wang W, Meng Y, Wang J, Qu S. An optical-transparent metamaterial for high-efficiency microwave absorption and low infrared emission. J Phys D Appl Phys. 2020;53(13): Article 135109.

[34] Datta RS, Syed N, Zavabeti A, Jannat A, Mohiuddin M, Rokunuzzaman M, Yue Zhang B, Rahman MA, Atkin P, Messalea KA, et al. Flexible two-dimensional indium tin oxide fabricated using a liquid metal printing technique. Nat Electron. 2020;3(1):51–58.

[35] Zhou Q, Yin X, Ye F, Mo R, Tang Z, Fan X, Cheng L, Zhang L. Optically transparent and flexible broadband microwave metamaterial absorber with sandwich structure. Appl Phys A Mater Sci Process. 2019;125:131.

[36] Zhang J, Li Z, Shao L, Zhu W. Dynamical absorption manipulation in a graphene-based optically transparent and flexible metasurface. Carbon. 2021;176:374–382.

[37] Liang JC, Gao Y, Cheng ZW, Jiang RZ, Dai JY, Zhang L, Cheng Q, Jin S, Cui TJ. An optically transparent reconfigurable intelligent surface with low angular sensitivity. Adv Opt Mater. 2022;12(6):2202081.

[38] Ruan Y, Nie QF, Chen L, Cui HY. Optical transparent and reconfigurable metasurface with autonomous energy supply. J Phys D Appl Phys. 2020;53(6): Article 065301.

[39] Zhang XG, Sun YL, Huang Z, Jiang WX. A review of light-controlled programmable metasurfaces for remote microwave control and hybrid signal processing. Eng Rep. 2023;5(9): Article e12658.

[40] Shadrivov IV, Kapitanova PV, Maslovski SI, Kivshar YS. Metamaterials controlled with light. Phys Rev Lett. 2012;109(8): Article 083902. 23002746 10.1103/PhysRevLett.109.083902

[41] Zhang XG, Sun YL, Zhu B, Wang J, Zhao T, Jiang WX, Huang Z, Zhang Z, Cui TJ. Optoelectronic metasurface for free-space optical–microwave interactions. ACS Appl Mater Interfaces. 2023;15(18):22744–22751. 37116067 10.1021/acsami.3c02290

[42] Sun YL, Zhang XG, Huang Z, Zhu B, Jiang WX, Zhang Z, Cui TJ. Remotely controlled laser-programmable microwave metasurfaces. Adv Opt Mater. 2024;12(16):2303139.

[43] Wang HB, Cheng YJ, Chen ZN. Dual-band miniaturized linear-to-circular metasurface polarization converter with wideband and wide-angle axial ratio. IEEE Trans Antennas Propag. 2021;69(12):9021–9025.

[44] Zuo Y, Rashid AK, Shen Z, Feng Y. Design of dual-polarized frequency selective structure with quasi-elliptic bandpass response. IEEE Antennas Wirel Propag Lett. 2012;11:297–300.

