# Supplementary material for: Intelligent Transmissive Microwave Metasurface with Optical Sensing and Transparency
Source: Research (Wash D C). 2024 Oct 21;7:0514. doi: 10.34133/research.0514 (PMC11491669; doi:10.34133/research.0514)
Supplement: Supplementary 1 — Notes S1 to S3 Figs. S1 and S2 [file research.0514.f1.docx]

**Supplementary Materials**

**Intelligent Transmissive Microwave Metasurface with Optical Sensing and Transparency**

Ya Lun Sun^1^, Xin Ge Zhang^1^, Zhixiang Huang^2^, Han Wei Tian^1^, Tie Jun Cui^1,4*^, and Wei Xiang Jiang^1,3,4*^

^1^State Key Laboratory of Millimeter Waves, School of Information Science and Engineering, Southeast University, Nanjing 210096, China

^2^Information Materials and Intelligent Sensing Laboratory of Anhui Province, Anhui University, Hefei 230039, China

^3^Purple Mountain Laboratories, Nanjing 211111, China

^4^Institute of Electromagnetic Space, Southeast University, Nanjing, 210096, China

^*^Address correspondence to: wxjiang81@seu.edu.cn, tjcui@seu.edu.cn

Ya Lun Sun and Xin Ge Zhang contributed equally to this work.

**Note S1. The** **experiment of the optical transmittance**

The optical transmittance of our metasurface sample was measured by using the Shimadzu UV-VIS spectrophotometer UV2600 with a wavelength range from 190 to 900 nm. This spectrophotometer is a commonly used instrument for accurately measuring the transmission or absorption of a sample in different spectra. In measurement, the sample needs to be placed in the sample chamber and covered with a lid to prevent interference of external light, and the experiment should be conducted in a dry environment for accurate measurement. Due to limited space of the sample chamber that requires the area of the tested film to be within 4 cm × 4 cm generally, a metasurface unit sample was cut out to be measured instead of the whole sample. And then, the unit sample was cleaned carefully to avoid dust and liquid as much as possible to prevent affecting light transmittance. After preheating the spectrophotometer, we selected the spectral range and let it calibrate automatically. Then we placed the unit sample vertically on the optical path to reduce optical reflection. At last, we set the spectrophotometer to scan the transmittance within the configurated spectral range and plot it as a curve.

**Note S2.** **Measurement of the response time of the metasurface**

The photoresistor used in our metasurface is made of cadmium sulfide (CdS). To measure the response time of the metasurface, we connect the photoresistor in series with a resistor into a circuit to measure its voltage waves by using the oscilloscope (**Figure S1a**), since the photoresistor cannot generate voltage, unable to be measured directly. Then we use an arbitrary waveform generator (AWG) to control the light source to generate varying light intensity signal (square wave changing between 0 and 8010 lux) to illuminate the photoresistor. The measured results are shown in **Figure S1b**. When the light source becomes bright fast, the resistance value can quickly decrease, with a response time of millisecond (ms) level. While the light source changes from bright to dim, the resistance value increases slowly and the response time is about 1 s. When the frequency of square-wave light intensity is 50 Hz, value of the photoresistor remains unchanged at 50 Ω. Thus, if the light intensity changes too quickly, the resistance value will remain at its minimum value. Therefore, when using rapidly varying light intensity signals for information transmission, the microwave channel can still remain the closed state, without affecting the function of the metasurface.


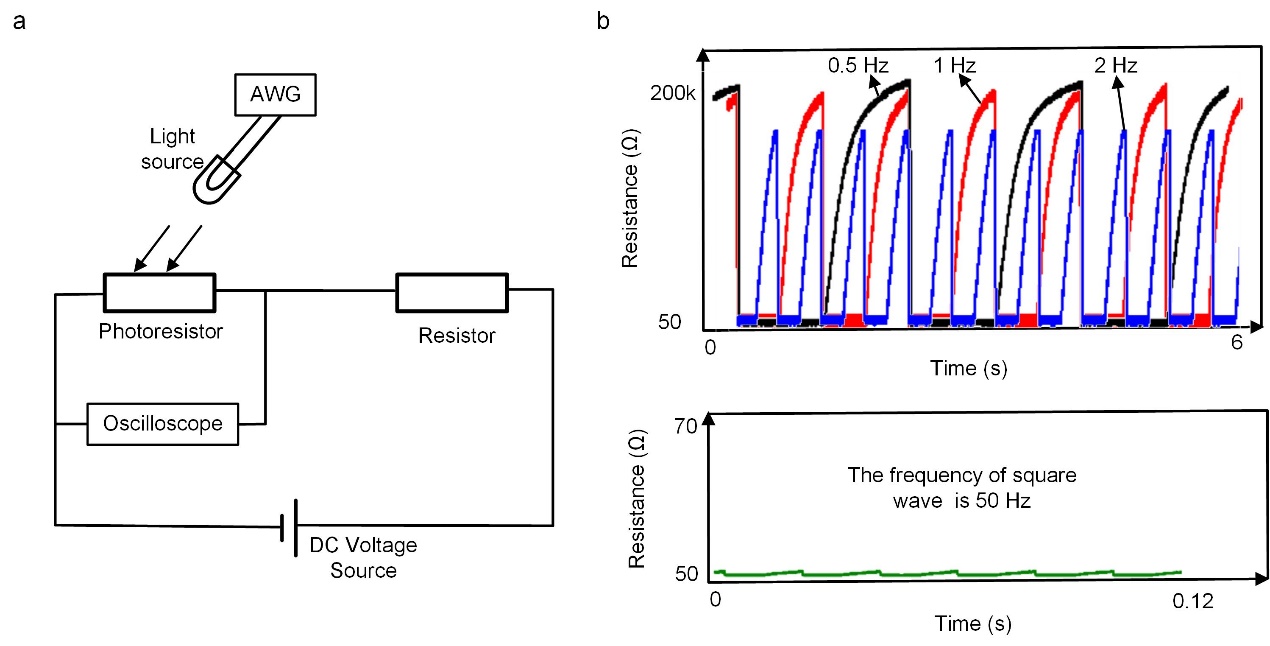


**Figure S1.** **Measurement of the response time of the metasurface.** (a) Schematic diagram for measuring the response speed of photoresistors. (b) Measurement results of the response time.

**Note S3.** **Experiments of using optical channel to transmit information**

We conducted experiments using optical channel to realize information transmission based on light intensity signals as shown in **Figure S2**. The optical information transmission module is located on the right side of the metasurface sample. The field programmable gate array (FPGA) can generate control waveforms according to transmitted data, and is connected to the digital to analog converter (DAC), thus driving the light source to generate light intensity signals with data. The receiving module is located on the left of metasurface sample, composed of a photoelectric detection circuit and an oscilloscope. The photoelectric detection circuit consists of a photodiode, a transimpedance amplifier, and a voltage amplifier, able to detect and recover the light intensity signals passing through the metasurface. Then the oscilloscope can display the received data signals composed of multiple waveforms which can be recovered to digital information, as shown in the left insets including waveforms of “0” and “1” (see our previous work Refs. 7 for more details). Therefore, the experiments prove that the optical channel of the proposed metasurface can be used to transmit information based on light intensity signals.


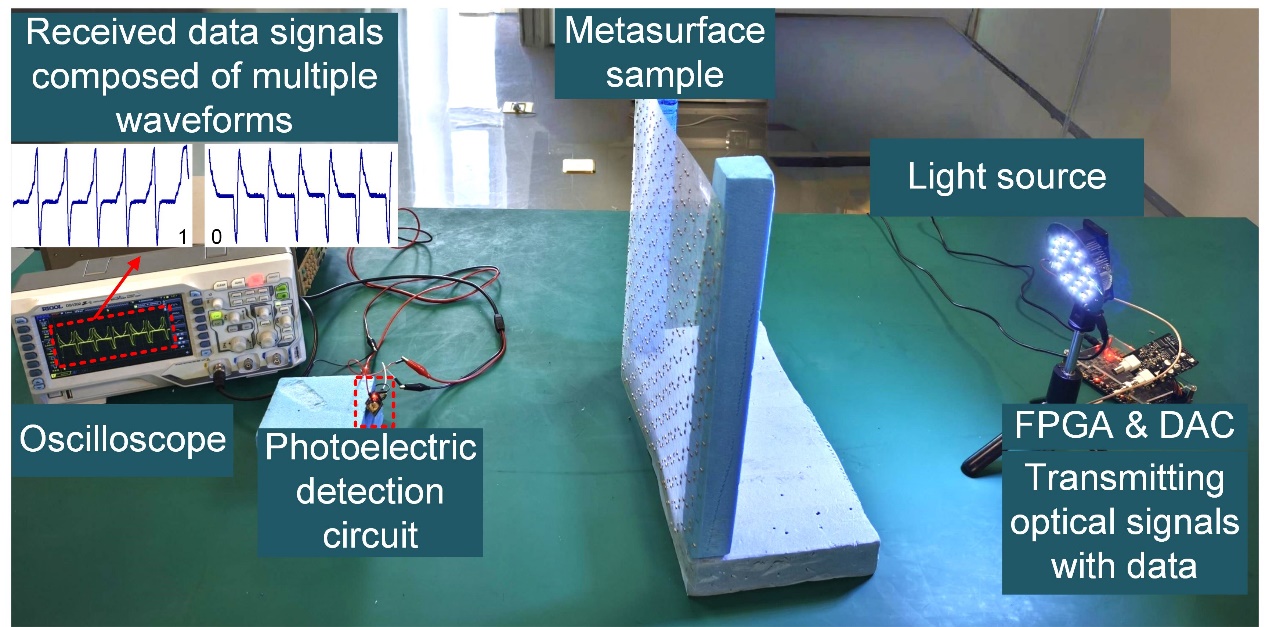


**Figure S2**. **Experimental scenario of using optical channel to transmit information**. The optical information transmission module composed of the FPGA, DAC and light source is located on the right side of the metasurface sample. The receiving module is located on the left, composed of a photoelectric detection circuit and an oscilloscope.
